# Supplementary material for: Development and Effect Evaluation of an Action-Oriented Interdisciplinary Weaning Protocol for Cuffed Tracheostomy Tubes in Patients with Acquired Brain Injury
Source: Healthcare (Basel). 2024 Feb 16;12(4):480. doi: 10.3390/healthcare12040480 (PMC10887695; doi:10.3390/healthcare12040480)
Supplement: Supplementary file 1 [file healthcare-12-00480-s001.zip › healthcare-2811797-supplementary.pdf]

Supplementary Materials

Table S1. Hazard Ratio for pneumonia before and after implementation of the interdisciplinary weaning protocol

**Variable**	**Adjusted HR (95%CI)**
Weaning protocol	
• Following IWP	0.899 (0.583;1.385)
• Before IWP	Ref.
**Sex**	
• Men	2.377 (1.373;4.116)
• Women	Ref.

Abbreviations: IWP = Interdisciplinary weaning protocol; HR = Hazard ratio. Right censored 120-days of hospitalization or at discharge, whichever came first.
